# Supplementary figures and images for: Unfolding the mitochondrial genome structure of green semilooper (Chrysodeixis acuta Walker): An emerging pest of onion (Allium cepa L.)
Source: PLoS One. 2022 Aug 30;17(8):e0273635. doi: 10.1371/journal.pone.0273635 (PMC9426943; doi:10.1371/journal.pone.0273635)

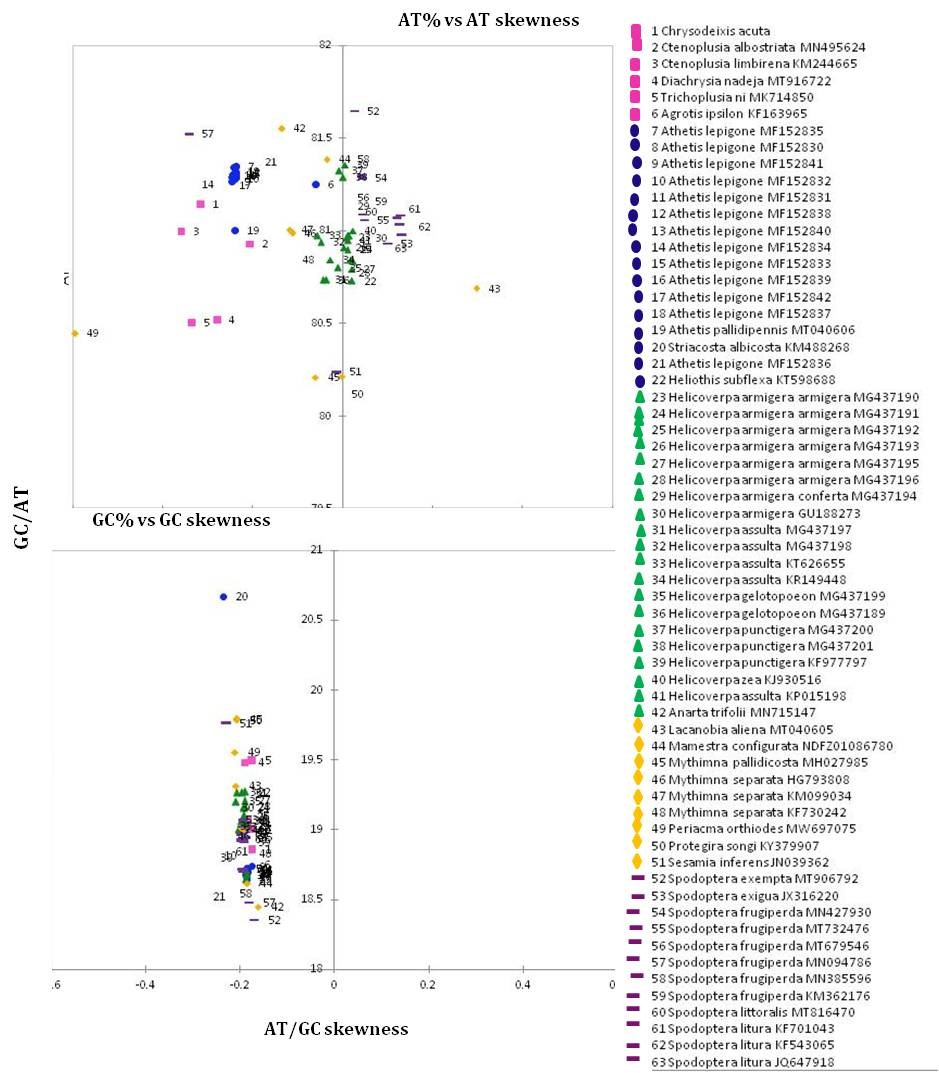

Supplement: S1 Fig — Values are calculated on J-strands for full length of mt genomes. The X-axis provides the skews values, while the Y axis provides the A+T/G+C values. Names of species are colored according to their taxonomic placement. (TIF) [file pone.0273635.s001.tif]

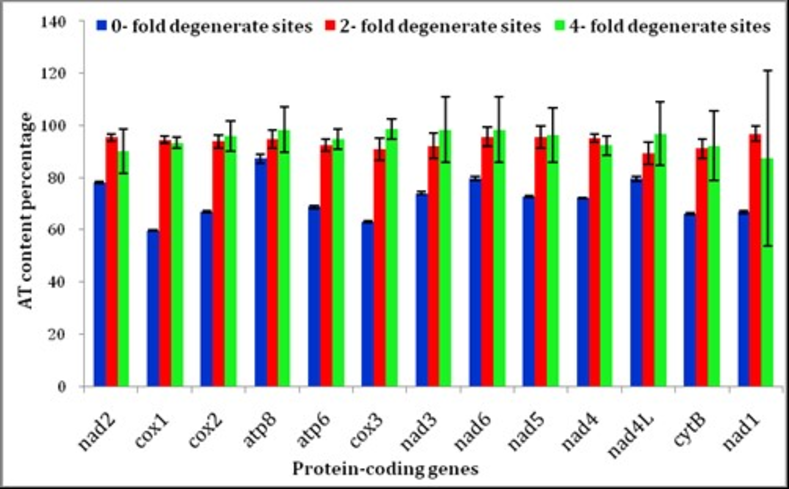

Supplement: S2 Fig — The black line with short line on the top of each bar represents the standard deviation value (SD). (TIF) [file pone.0273635.s002.tif]

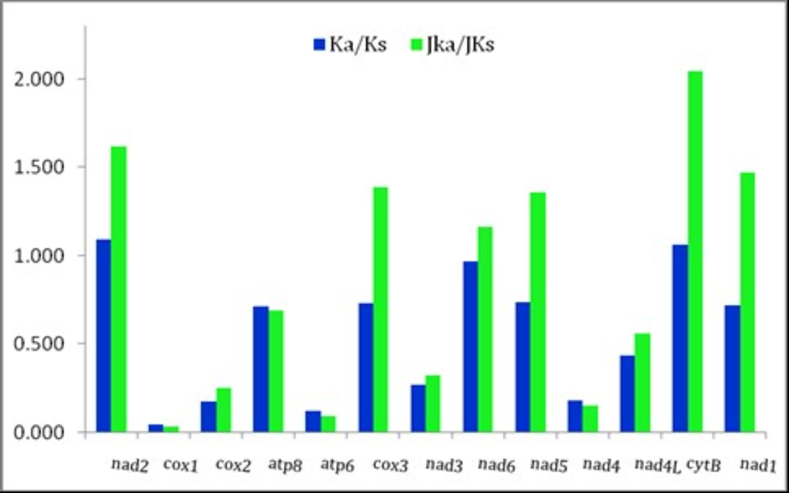

Supplement: S3 Fig — (TIF) [file pone.0273635.s003.tif]
