# Supplementary material for: Unfolding the mitochondrial genome structure of green semilooper (Chrysodeixis acuta Walker): An emerging pest of onion (Allium cepa L.)
Source: PLoS One. 2022 Aug 30;17(8):e0273635. doi: 10.1371/journal.pone.0273635 (PMC9426943; doi:10.1371/journal.pone.0273635)
Supplement: Attachment — Submitted filename: Response to Reviewers.docx [file pone.0273635.s004.docx]

| **S. No.** | **Reviewer comment** | **Author Response** |
| --- | --- | --- |
| **Reviewer 1** | | |
| 1 | How many samples were selected? The stage of the larval? And the mitochondrial genome was sequenced only once? | Laboratory reared fully grown fourth instar larvae of *Chrysodeixis acuta* was used for the mitogenome analysis. Single sample was sequenced. As per the reviewer’s suggestion, necessary changes has been made in the materials and methods section of the revised manuscript. |
| 2 | The quality of the figures is not good and need improvement. | Quality of the figures are improved and included in the revised manuscript. |
| 3 | The scientific name of the species should be italicized and the format of references is inconsistent | Scientific name of the species were italicized and the formatting of the references have been done as per the journal norms. |
| 4 | The English should be further improved | As per the reviewer’s suggestion, necessary changes has been made in the revised manuscript. |
| **Reviewer 2** | | |
| 1 | I would only suggest a widening of literature references, also considering some more recent works that came out, and hence a deeper discussion. | As per the reviewer’s suggestion, discussion section has been elaborated with recent publications in the revised manuscript. |
| 2 | Revise for minor typographical and grammatical errors | Both minor typographical and grammatical errors has been rectified in the revised manuscript. |
